# Chicago Medical Society: Meeting June 21

**Published:** 1886-09

**Authors:** 


### SOCIETY REPORTS.

CHICAGO MEDICAL SOCIETY.

*Regular Meeting, June 21, 1886.*—THE PRESIDENT, E. J. DOERING, M. D., in the chair.

PROFESSOR F. E. WAXHAM read a paper entitled

INTUBATION OF THE LARYNX AS A SUBSTITUTE FOR TRACHEOTOMY, WITH A REPORT OF 83 CASES.*

PROFESSOR D. A. K. STEELE, in opening the discussion, said that of course the principal interest to the surgeon centers in the question of the performance of tracheotomy in croup or diphtheria. He had looked up some of the older statistics, in addition to those given by Dr. Waxham. The French seem to have led the way in the practice of tracheotomy. Trousseau, in 1858, gave the statistics as about 25 per centum of recoveries, and from later reports tracheotomy does not seem to have gained much. Dr. Solis-Cohen, of Philadelphia, in 1883, recorded some 5,000 cases, and his per centum was the same as given twenty-five years before by Trousseau, about one in four. More recent statistics show some improvement in tracheotomy; for example, Dr. Jacobi, of New York, published 1,000 cases, in which he had a little better success, if he remembered correctly, than those reported by Solis-Cohen. A few days ago he saw a report of seventy-seven cases from the Boston City Hospital, with a

* Professor Waxham's paper appeared in full in our August issue, but the discussion was crowded out by want of space.

recovery of twenty cases, about 25 per centum. In Chicago the statistics, as given by Dr. Waxham, are 306 cases with a recovery of about 19 per centum; these are the most recent statistics he had found. Of course, in regard to the report of cases of tracheotomy, we have to bear in mind a very practical point—that is, that the unfavorable cases are not all reported. The majority of physicians who do a few operations and have not a successful result do not record their cases. In New York city, some twelve years ago, a surgeon reported 67 cases with 13 recoveries. He made operations on 100 additional cases before he had another successful one ; so there are curious mixtures and changes in the statistics of different operators.

Another point is that a good many surgeons report as successful, cases that recover from the operation, but that die of some other affection or sequelæ, as the case reported by Dr. Waxham dying from albuminuria. All these cases are reported by tracheotomists as recoveries; but they are not recoveries from the diphtheritic croup. These points must be borne in mind. When we sum up, the statistics to-night would show a recovery of 27 per centum, which certainly seems to be a very favorable showing. In addition to these cases reported by Dr. Waxham, Dr. O'Dwyer has of late recorded 25 cases with a recovery of 24 per centum; taking all those cases into considerstion, the Chicago record is ahead of the New York record by 3 per centum. In regard to the Boston City Hospital, where they had 25 per centum of recoveries, we should bear in mind that those cases presumably have much better surroundings than we have in Chicago, or in general practice. There they have the best of nursing and immediate attention in case any mishap occurs, which in some cases in private practice might go unnoticed for many hours. With the favorable surroundings so far as the operation and the after-treatment are concerned, the reported recoveries of 25 per centum are as favorable as we could expect.

Intubation is a comparatively new operation, but with the improved tubes—as by enlarging the calibre of them (for a serious defect in the construction of these tubes has been that the lumen was too small)—the percentage of recoveries would be increased. There would be less danger of rolling up the detached pieces of matter, and he thought that expectoration would occur more freely. It is supposed to be necessary to have the tube extend down near to the bifurcation of the trachea, so as to prevent the curling pieces of membrane blocking up the opening, but he thought they were longer than is necessary. The principal obstruction, we know, is at the larynx; the membrane below the trachea is thinner, smaller, and is not likely to curl up in this way. Theoretically the tube should be long, practically it might be shortened, and with the improved tubes we will get still better results.

A point in favor of intubation over tracheotomy is the rapidity with which it can be done, taking only the fraction of a minute to insert a tube in the hands of an experienced operator. We might also take into account the greater comfort ot the doctor after the operation; the feeling of security with the laryngeal tube. He is constantly in dread after tracheotomy, watching and waiting, and seeing the little patients upon whom he has operated die one after another. Then the relief from dyspnœa seems to be quite as great after intubation as after tracheotomy. The relief from urgent symptoms is just as marked with the introduction of the laryngeal tube as with tracheotomy. In case we find intubation does not afford permanent relief it is no bar to tracheotomy subsequently, although where intubation does not relieve dyspnœa, tracheotomy will not relieve it; where stenosis is not relieved by the introduction of the tube, it does not relieve the dyspnœa.

PROFESSOR W. E. CASSELBERRY said his experience either with intubation or with tracheotomy has not been very extensive, but from it and from what he had gleaned from the literature of the subject, including the valuable paper presented to-night, he was very favorably impressed with the operation ot intubation. The most favorable statistics of tracheotomy are those of not only the French, but more particularly of the large hospitals of France and Germany. In these institutions they have the fullest command of the patients in every way; the greatest attention can be paid them and the operation can be and is performed early. The most favorable statistics are those of Plenio (Königsberg)—and of all the cases that were taken to the hospital but five escaped tracheotomy, showing that almost all were subjected to the operation. The case of Dr. Ingals, in which the membrane was removed from one of the bronchial tubes, was a most brilliant one, but then intubation does not prevent tracheotomy from being performed afterwards. The forceps for the removal of the membrane through the mouth, which the author has exhibited this evening, is new; he has not much confidence in it, but perhaps it may be of value. In reference to the length of the tube, somebody has proposed that it should be shorter. He thinks it is the length of the tube which has rendered this operation successful over the operation of some eighteen or twenty years ago. It was invented by a French surgeon at that time, but the tube was only about three-fourths of an inch long, sufficient only to reach into the larynx, where it excited irritation, and the operation was discarded as of no value; so that he thinks in the length of the tube we have an important feature. In reference to the introduction of the tube, if it is possible to practice on the cadaver it would be of great value; a minute is a long time when you are introducing a tube into the larynx of a child who is suffocating; you gag the mouth, introduce the forefinger as a guide to the larynx, follow it by the tube; the child stops entirely his feeble respiration, in a few seconds he becomes black in the face, and you are very anxious either to get the tube in or your finger out. If we bear in mind the instructions of the author of this paper to pass the tube under and not over the forefinger as a guide, no doubt we will be successful. It should be remembered that the curve into the trachea is a right-angled curve, so you must turn acutely and not obtusely or you will enter the œsophagus and not the trachea. The food of the patient is a most important point; when the tube is in liquids will frequently run into it and excite irritation.

DR. A. B. STRONG said: Before coming here he was prepared to indorse this operation to a great extent. He did not suppose the statistics were so favorable, but he was glad to find they are so. He has had quite a number of cases of intubation and of tracheotomy, and he must say that he much prefers the introduction of the tube. However, not every case is adapted to the intubing. He thinks there are many cases where tracheotomy would be far preferable. We can feed a little patient after tracheotomy much better than after intubation of the larynx; to introduce a stomach tube would require the attendance of the physician several times a day. He has not yet given a fair trial to introducing the food into the rectum. He would not want a little patient that had suffered for three or four days before the operation to go without food. He likes to feed his patients well, and certainly would not wish to starve them. There are three or four cases that have given him a good deal of information. The operation is new; we do not know all there is about it. One of the first cases that he had of true diphtheria, where intubing was done, was a little boy 4 years of age, taken sick five days before he saw him.. The physician in attendance had given the child up, and Dr. Strong was called in. The little fellow was as near dead as any patient he had seen, but he would not have recommended tracheotomy in that case. The neck was badly swollen. The patient was cold, clammy, pulseless and cyanosed, with his pupils widely dilated and apparently just on the verge of dissolution, mainly from profound blood-poisoning. He introduced the appropriate tube, and much to his own surprise he nearly strangled him; indeed, for a moment he thought him dead. He drew the tube out and the child breathed a little better. It occured to him that he might have crowded the membrane down. He introduced the tube again, with the same result; he was nearly strangled. He tried a smaller tube, but each time the tube went in the child nearly died. He laid him on the bed and surrounded him with dry heat. He gave him brandy hypodermically. He did not suppose the child would live more than an hour, but to his surprise he received a message by telephone that he was alive and doing well, ten hours afterward. He went to see him and found him with a good color, taking considerable nourishment. He called again in six hours just in time to see the child die. He had the privilege of post-mortem, and found exactly the condition of affairs he had anticipated. At the lower portion of the trachea he found a complete cast of that membrane crowded down. This case had been going on about five days when he saw it, and this membrane had been loosened in the meantime and so crowded down.

Another case was of a good deal of interest. A little girl eight years of age had a mild attack of diphtheria four days prior to his seeing her. Two days before he saw her the larynx first became involved, and gradually grew worse. The case was one of the most favorable for operation that he has seen. He introduced the largest sized tube and it remained in position for three days, and within 24 hours the lungs cleared up very nicely; at the end of three days and two hours the tube was removed with comparative ease, and he noticed that the child did not breathe as freely as he supposed she would. He remained about an hour, and during that time dyspnœa set in and the child nearly choked to death. He put in the one next in size and the symptoms were instantly relieved. The child wore that tube two days, when it died from bronchitis.

That case gave him this idea: the tube first introduced was as large as the parts would readily take in—it required a little force to introduce it—and it occurred to him that the tube was larger than was necessary. It remained there three days and two hours, squeezing the blood out from these parts, and when it was removed the sudden reaction caused the œdema of the larynx. The child wore that tube five days and a few hours, and until the last twelve hours of its life was getting along very nicely, except that it did not take the amount of nourishment he considered necessary. This operation is brilliant, and has a future before it, and he can heartily endorse it to-day. He is perfectly willing to try it, and would prefer, in case of one of his own children, intubing the larynx to opening the windpipe.

PROFESSOR CHRISTIAN FENGER said: The subject is a most important one. As to the history of the operation, he would say that it is not a new one. A French surgeon, Loiseau, invented it in 1858, and to him belongs the idea. The instruments were, in the principle, about the same which we use now; there have been modifications in details only. Tubage was tried in France by Gros, but seems not to have found favor, although Bonehut recommended it without having used it himself. It was tried in Vienna by Weinlechner, who recommended it. It was condemned by Steiner and Ziemssen, and given up again. In reference to what was said about patients not swallowing at all for the first twenty-four hours, it seems to him it would be difficult to keep little children from drinking milk, or water perhaps, milk anyway, because they are always thirsty. There will thus be danger from foreign body—pneumonia (Schluck pneumonie). It might be that feeding bottles will obviate that danger. A second point is this: He saw a child treated by intubation die from loosened membranes closing the bifurcation. Whether the membranes were entirely loose or not, or loose above and attached below, is not materially important. The case convinced him that he would have felt safer with tracheotomy, because loosened membranes can be reached easier through the tracheotomy tube than through the larynx. As to the statistics, it is quite certain that the tracheotomy statistics are a little better than have been given here to-night. In the large hospitals in Germany the recoveries reach up, in the last few years, to 31 per cent. At the same time, when intubation shows 27 per cent, that is in itself reason enough for a trial. All these points are not exactly exclusively in favor of intubation, but like Dr. Strong, he should first try the tube, if it was his own child, and watch the patient incessantly, with the tracheotomy instruments on the table to be ready at any time when needed to resort to tracheotomy.

PROFESSOR F. O. STOCKTON was in favor of intubation.

PROFESSOR R. G. BOGUE said that there is much to recommend intubation he believes. The testimony we get is certainly enough to recommend the operation and to establish it as a practical procedure and one worthy of trial. It affords probably as much relief for the urgent symptoms, the one for which tracheotomy would be resorted to, as tracheotomy would; and the length of time that the tube is to be worn will be shorter than the tracheal tube. These are reasons why it should. From the pressure of the tube upon the membrane the presence would be likely to cause some disintegration of the membrane, and in that way the membrane would be taken away or gotten rid of sooner than without the pressure. The only objection—at least the one which occurs to him from what little he has seen and what we hear—is the matter of fluids passing through the tube into the trachea and into the respiratory passages while attempting to feed the patient. That is an annoyance and probably a detriment. It also, he supposes, will deter children from trying to take fluids. Little children soon learn to avoid things which hurt them, and he should think that it would be a factor in preventing the free feeding of children. Now it may be that the introduction of fluid into the air passages may not be harmful, for as recently as that day he saw in some report from New York the recommendation that in case of tracheotomy, where there is obstruction below the tube, to pour fluid, by teaspoonful, for instance, into the tracheotomy tube, letting it run down into the air passages, for the object of liquefying and softening the membrane and getting rid of it in that way. That, he thinks, is recommended by Dr. George F. Shrady, who had practiced it recently with apparent benefit.

The question of introduction of fluid into the air passage may not be a tangible objection. But we would perhaps prefer to select the kind of fluid we would put into the trachea instead of milk or beef tea, or anything of that kind. The operation of tracheotomy has been resorted to for a great many years, with varying success by different operators and in different localities. Trousseau years ago reported his success as about one in three, or three to four, from a very large number of cases. In the cases we have heard this evening, as compared one with the other, tracheotomy hardly holds its own. The number of cases in tracheotomy no doubt are not all reported, but a fair proportion of them probably have been reported. While tracheotomy is certainly of great benefit, and has proved itself to be such in this class of diseases, it is subject to its disadvantages and annoyances; but so also has intubation its annoyances, both to the patient, the friends and the doctor.

PROFESSOR C. T. PARKES said he had nothing but words of commendation as far as Dr. Waxham is concerned, for his persistence in presenting this method of O'Dwyer to the profession of this city. He himself has nothing to say about intubation, having had no experience, and never having seen a tube used. He has had a little experience in tracheotomy, and so far as his personal experience goes he will stick to that until he has reason to change it. His experience extends over thirty-one cases of tracheotomy. He had the honor to report to this society on previous occasions the first fifteen cases; eight recovered and seven died. He has collected the names and residence of sixteen more, and of these ten have recovered and six died. That is a pretty fair showing. He cannot explain, and does not pretend to explain this percentage of recoveries. You will find in the literature of tracheotomy throughout the world that some operators have good fortune in that respect. He does not think it an unusual or peculiar fact that the doctor relates about relief given to the patient when you open the trachea after stenosis; cases in which the patients are unconscious, black in the face, and are revived by the introduction of the laryngeal tube; such a result is to be expected. He noticed by the report that very few, if any, of these cases recover after intubation. He knows he has seen just such cases recover after tracheotomy. He has never had any trouble from the swallowing of fluids or food of any kind. He has seen the fluids come out of the opening of the tube, and has had the physician in charge reporting that we had unfortunately made an opening in the œsophagus; those cases have never given trouble, and in a few days subsided. There is one peculiarity about these cases of intubation, that Dr. Bogue has explained, the pressure of the tube causing an early disappearance of the membrane. There have been no cases that he has seen in tracheotomy where the tube could be removed under six days. In two cases it was three or four months before the tube was removed. He has never seen the trachea opened, so far, without seeing false membrane come out of it. If he were enabled to remove the tracheotomy tube inside of six days or a week, he should think that to be a case in which no membrane was found in the trachea, and one, in all probability, in which if he had waited a few days the patient would have gotten well without any operation. There is something peculiar and interesting in the ages of these sixteen cases, the youngest being 3½, the oldest 37 years of age. He thought the cases in which stenosis has been complete, and required an opening of the trachea, have been very few in the adult. One of his operations was upon a woman after she had been sick some four or five days with a bad case of pharyngeal diphtheria. At the time he saw her she was absolutely suffocating, and he was called for the purpose of performing tracheotomy. He did the operation under very poor surroundings and yet the patient recovered. The tracheal opening became diphtheritic. Another case was 32 years old, and the patient died on the table. This accident has happened in quite a number of cases. He never had any trouble with the children, and certainly expected less with the adults, as in the latter the operation is much more simple than in the child. This patient stopped breathing before the trachea was reached, and after it was opened it was impossible to restore respiration. As far as he is concerned, he can say nothing about intubation. He don't know whether or not it is such an easy thing to intube the larynx. He thinks he has cognizance of three cases not in the doctor's list that proved fatal, in which some difficulty was met. One three years old died the day after. One seven, in which no relief was given, as the membrane seemed to be pushed ahead of the tubes; and one in which, no relief being given, the attempt to remove was complicated by the child swallowing it and being buried with the tube in its stomach. Another, a young lady seventeen years old. This patient recovered; she was relieved immediately after the introduction of the tube, and rested very comfortably until the next day, when she complained of a pain in her stomach. In the evening of that day she passed the tube from the rectum. He allows others to judge of the necessities requiring its use, and so don't know that he can agree with these gentlemen and say the operation is simple. We know what tracheotomy amounts to, and he is sure he would not feel right about it, that he should be haunted with the idea of not having done his whole duty, if he left a child seven years old go without doing tracheotomy where he felt satisfied after this tube was introduced there was no relief given.

He thinks tracheotomy will always have a place. He has been asked many times how he came to have this record of tracheotomy, that he thinks it is due him to say one thing more; he does not know how to explain it. He believes no other operation for tracheotomy should be done but the high operation. The trachea should be opened at the highest ring, and if necessary divide part of the cricoid, as he has done, or all of it. Certainly the after care has a great deal to do with the recovery, and he does not believe there are any persons so wanting in intelligence but if you give them instructions carefully they will carry them out. So far as his experience goes, the surroundings have made no difference, even if it be a house of only one room with the whole family living in it and several other animals besides. He thinks the room should be kept at a temperature above 80; there should be no attempt to fill it with moisture; the room should be so hot as to be decidedly uncomfortable for all the friends for the first twenty-four hours. During this time —twenty-four hours—the inner tube of the trachea should be removed every hour, whether the patient seems to need it or not, because that is the time when the deep-seated trouble in the organs below starts. After that time he does not believe the tube should be removed oftener than seems absolutely necessary, and he does not believe that mucus can be prevented from drying by the introduction of foreign bodies. Every time the tube is removed it should be reintroduced damp, or wet in a mild solution of soda; and occasionally, without taking out the inner tube, drop into the trachea a few drops of this same fluid, so as to keep the end of the tube as moist as possible. No foreign bodies of any kind should be introduced into the trachea after you are quite certain all the pieces of membrane have been detached and removed at the time of the operation. The introduction of such an innocent little thing as a feather or sponge, he is satisfied, does more harm than good. He is willing to admit that in one case which showed every sign of recovery, the tube was moist and the cough was moist, and he had every reason to believe it would get well, but owing to over-anxiety he kept poking feathers into the trachea and had the friends do the same, until an inflammation of the trachea proper was produced around the tube, which he is satisfied was the cause of the fatal issue. He does not think there should be any interference; the least possible interference is the best.

DR. H. T. BYFORD said he wished to call attention to one fact; that is, in the statistics of hospitals tracheotomy shows about as favorably as intubation, although in private cases the safety, simplicity, and absence of formidableness of intubation makes it already the popular operation. As he has said before this, tracheotomy is an operation for hospital practice, where the difficulties can be overcome, and where its just claims for precedence in particular cases must always obtain consideration.

PROFESSOR F. E. WAXHAM, in closing the discussion, said he believes every tracheotomist will entertain an opinion in regard to tracheotomy according to the success which he has attained. If a physician has saved every patient upon whom he has operated, he will feel that the operation is good enough, and will be perfectly satisfied; if he has saved a large proportion of his cases he will be loth to accept anything new; but the great majority of physicians, who have either met with unvarying failure or inferior success, will have a poor opinion of the operation. English writers seem to be losing faith in tracheotomy, for Holmes, in his "System of Surgery," says: "English medical men seem very generally to incline to the opinion that the operation, if not to be recommended, is at least justifiable, but to be successful it must be performed at a very early period of the attack." Referring to Professor Parkes' remarkable record of tracheotomy, he can not help feeling that his cases could not have been so unfavorable. He cannot believe that he had many young infants to operate upon. He would like to ask the age of the youngest patient?

PROFESSOR PARKES: Three and a half years.

PROFESSOR WAXHAM: That explains why his record has been far more successful than that of many physicians, and he believed that if tracheotomy were performed upon every case without reference to age, condition, or type of disease, we would not save more than one out of every ten or twelve. Many physicians select their cases; indeed, he knows of some who *will not* operate upon a child under three years, and of others who will not operate upon diphtheritic cases on account of the known fatality occurring after tracheotomy in these cases.

In regard to the difficulty of performing intubation, he would say that any of you who attempt the operation upon a child without practice upon the cadaver will certainly be sorry for it. He has known of four physicians who have failed completely. In regard to the patient to whom the last speaker referred, the young lady with the tube in her stomach, that case was not in his record, for the patient was not his.

PROFESSOR PARKES has stated that after tracheotomy, if he were able to remove the tube in less than six days he would have concluded that there could have been no membrane within the larynx, and that if he had waited a few days longer the patients would have recovered without the operation! A natural inference would be that in these cases of intubation, because the tube has frequently been removed on the third, fourth or sixth day, that there could have been no membrane present and that the operation was unnecessary. In reply he would state that in every case coming under his observation false membrane has been expelled when the tube was introduced. The reason why the laryngeal tube can be removed earlier than the tracheotomy tube is that more or less membrane is detached when the tube is first introduced, and second, the pressure of the tube and the frequent coughing assists in dislodging the membrane; while after tracheotomy little or no air passes through the larynx until after the removal of the tube, and there is nothing to cause the dislodgement of the membrane.

In regard to feeding the patient. Many of his patients have taken abundance of nourishment without difficulty. Occasionally, however, there will be one that will not take a sufficient amount. It will depend a great deal upon the size of the tube. If we use a tube larger than is appropriate for the age of the patient, it will not always fit perfectly into the cavity of the larynx, and consequently the epiglottis will not close accurately over it. In a case such as Dr. Strong has referred to, where the membrane is pushed down ahead of the tube, the trachea forceps would be very useful in removing it.

There are a great many dangers from tracheotomy that are never met with from intubation; but we rarely hear of them. He had learned of one physician who attempted a tracheotomy on a child four years old. Cutting down hastily he severed large blood-vessels, and losing his head at the sight of the copious hæmorrhage, at once sewed up the wound, while the child, almost with his last breath, wanted to know if they were going to cut him any more. Another physician, in cutting down hastily in order to open the trachea, missed it, and accidently severed the carotid artery. Indeed, the history of tracheotomy is replete with horrors, but we do not hear of them. In the hands of experienced surgeons, who are cool and collected, the operation may be a simple and safe one; but in the hands of the inexperienced and the nervous, performed, as it frequently is, in the dead of night, it then becomes a formidable, yes, a dangerous procedure. Intubation, on the other hand, can be performed quickly, in a very few seconds, and without the dangers attendant upon tracheotomy.

---

CHICAGO MEDICAL SOCIETY.

